# Single-cell sequencing in primary intraocular tumors: understanding heterogeneity, the microenvironment, and drug resistance

**DOI:** 10.3389/fimmu.2023.1194590

**Published:** 2023-06-09

**Authors:** Lin-feng He, Pei Mou, Chun-hui Yang, Cheng Huang, Ya Shen, Jin-di Zhang, Rui-li Wei

**Affiliations:** ^1^ Department of Ophthalmology, Changzheng Hospital of Naval Medical University, Shanghai, China; ^2^ 92882 Troops of the Chinese People’s Liberation Army, Qingdao, China

**Keywords:** single-cell sequencing, retinoblastoma, uveal melanoma, heterogeneity, microenvironment

## Abstract

Retinoblastoma (RB) and uveal melanoma (UM) are the most common primary intraocular tumors in children and adults, respectively. Despite continued increases in the likelihood of salvaging the eyeball due to advancements in local tumor control, prognosis remains poor once metastasis has occurred. Traditional sequencing technology obtains averaged information from pooled clusters of diverse cells. In contrast, single-cell sequencing (SCS) allows for investigations of tumor biology at the resolution of the individual cell, providing insights into tumor heterogeneity, microenvironmental properties, and cellular genomic mutations. SCS is a powerful tool that can help identify new biomarkers for diagnosis and targeted therapy, which may in turn greatly improve tumor management. In this review, we focus on the application of SCS for evaluating heterogeneity, microenvironmental characteristics, and drug resistance in patients with RB and UM.

## 1 Introduction

Retinoblastoma (RB) and uveal melanoma (UM) are primary intraocular tumors that mainly originate in the retina and uvea, severely impairing visual acuity and threatening patients’ lives (1, 2). Although rare, RB is the most common childhood intraocular tumor and tends to disseminate intracranially and distally. The survival outcomes of patients with RB differ substantially between different regions, with a survival rate of just 57.3% at 3 years in low-income countries, and tumor metastasis remains a major cause of death (3). Uveal melanoma (UM) is the most common intraocular tumor in adults, with a propensity to metastasize to the liver (50% of patients may develop metastases within 15 years) coupled with a high mortality rate (4). Meta-analyses have revealed that the median overall survival is 10.2 months for patients in whom metastasis has occurred, with an extremely poor curative rate (5). To date, enucleation, radiotherapy, and chemotherapy for metastatic intraocular tumors have not effectively improved prognosis among these patients (6). The exact mechanisms underlying the genesis and metastasis of intraocular tumors are not fully understood.

Tumor heterogeneity refers to the different phenotypic profiles and morphologies of distinct tumor cells, and the interaction between the tumor microenvironment (TME) and tumor cells has been shown to influence disease progression, drug resistance, tumor invasion, and prognosis (7). However, traditional bulk sequencing technology is performed using homogenized tissues, which can only provide overall information regarding the pooled cell population and cannot reveal the characteristics of cell heterogeneity. Unlike bulk sequencing, single-cell sequencing (SCS) strategies for genomic, epigenomic, transcriptomic, proteomic, and metabolomic analyses enable researchers to obtain accurate cellular and molecular information concerning individual cells. SCS strategies have revolutionized our understanding of the biological landscape and the dynamics of malignant diseases (8). Therefore, research performed at a single-cell resolution can provide greater insight into cell–cell communication and the heterogeneity of tumor cells. In this review, we summarize the recent progress in obtaining information on RB and UM via SCS, including insights into tumor genesis, heterogeneity, microenvironmental properties, and drug resistance.

## 2 Tumor heterogeneity

Cells in tumor tissues, which are characterized as diverse and functional diversity under different pathological conditions, are highly heterogeneous, which may influence the therapeutic response to targeted therapy and survival outcomes. The emergence of SCS technology has enabled researchers to evaluate the immense biological complexity of tumors. Liu et al. constructed a cancerous organoid model of RB using genetically engineered human embryonic stem cells with a biallelic mutation in the RB susceptibility gene *RB1*, which was highly consistent with primary RB tumorigenesis, transcriptomic characteristics, and genome-wide methylation (9). Single-cell RNA sequencing (scRNA-seq) analyses of the organoid model revealed four extra cell clusters when compared with human retinal organoids: RB cells, retinoma-like cells, unfolded protein response-related cells, and excessive cone precursors. The cone precursors expressed several cone precursor markers, such as ARR3 and RXRG, suggesting a potential cellular origin for RB. Further investigation using single-cell pseudo-time trajectory analysis confirmed that the maturing cone precursor was the cellular origin of RB in cancerous organoids. Notably, the PI3K-AKT pathway, a key cancer-related signaling pathway in RB tumorigenesis, is dysregulated and its activator, spleen tyrosine kinase (SYK), is significantly upregulated in retinal organoids, which could be the basis for the development of potential drugs targeting SYK (9). Collin et al. performed the first scRNA-seq and single-cell assay for transposase-accessible chromatin sequencing (scATAC-Seq) of primary RB samples from patients (10). A total of 8,086 cells from the two samples were sorted into 18 cell clusters, five of which (i.e., clusters 2, 8, 11, 12, and 14) were in the G2/M phase, and 13 of which (i.e., clusters 0–9, 11, 12, and 14) were identified as cone precursors. The researchers then performed a pseudo-time analysis of cone clusters and suggested that G2/M cone precursors (i.e., clusters 2, 8, 12, and 14) were the cellular origin of RB, corroborating the findings mentioned above. To further explore the molecular mechanisms that cause cone proliferation during the development of the human retina, researchers performed scRNA-Seq and scATAC-Seq of nine retinal samples and two RB samples. Two RB tumor-specific cone subclusters were identified, and each subcluster was characterized by the activation of individual upstream regulators and signaling pathways, leading to the dysregulation of p21 and p53 which further enabled the escape from apoptosis and cell cycle arrest. The authors also proposed that cellular apoptosis is mediated by p53 as the final compromised event in the two subclusters. These findings not only demonstrate tumor heterogeneity in RB, but also provide insight into the potential molecular pathways that could be targeted in the treatment of RB.

Yang et al. sorted 14,739 cells from two RB tumor samples into 10 clusters based on single-cell transcriptomic profiles, observing that the major cell types were cone precursors and RB cells (11). An analysis of the specific developmental trajectory of the RB revealed two subtypes of cone precursors (i.e., clusters 7 and 8) at branch point 2, with RB cells present after two branches. The cell trajectory was separated into five states based on the branches. State 3 launched the delamination of the RB, and state 5 was characterized by the high level of expression of cell cycle-related genes and a gradual shift in the malignancy process. In addition, *UBE2C*, which is abundant in state 5 of RB cell cluster 5, plays a crucial role in RB progression and represents a potential therapeutic target.

Tumor heterogeneity can also be examined from another perspective, focusing on the diversity of the expression and function of tumor subsets. To gain an insight into the intratumoral heterogeneity of RB, a previous group analyzed the single-cell transcriptome and whole exome of patients with RB (12). The authors noted that the principal cell types in RB were cone precursor-like (CP-like) cells and *MKI67+* cone precursor (*MKI67+ CP*) cells, with a few cells maintaining the features of normal retinal photoreceptor cells. Their analysis of RB phenotypes identified the C7 and C10 subtypes of *MKI67+* CP cells as more malignant, whereas CP-like cells were thought to reflect a transitional state between normal cells and *MKI67+* CP cells. RB samples presented large clonal heterogeneity and the malignant *MKI67+* CP cells exhibited greater changes in copy number. Importantly, both tumorigenic cell subpopulations and normal cells were present in RB, and the degree of malignancy in the tumorigenic cell subpopulations varied, which fully supports the intratumoral heterogeneity of RB.

UM is a highly heterogeneous malignant tumor. By performing clustering analysis on six freshly collected primary UM tumor samples, Pandiani et al. found that most cells could be grouped by tumor of origin, supporting the notion of intertumoral heterogeneity (13). Furthermore, unbiased clustering produced 12 clusters; among them, clusters 2, 4, 7, 8, and 10 were related to poor prognosis. Using single-cell regulatory network inference and clustering technology, they found that clustered regulons (*RELB, HES6, HSF1*, and *MYC*) are associated with a poor prognosis. Depletion of *HES6* (an enhancer of split family basic helix–loop–helix transcription factor 6) has also been shown to weaken disseminative, migratory, and proliferative abilities both *in vitro* and *in vivo* in primary UM. Furthermore, Sun et al. integrated the scRNA-seq dataset from 33 samples (19 patients with primary UM, three patients with metastatic UM, and 11 healthy controls); up to 222,075 cells were classified into 12 clusters, and the UM samples mainly consisted of melanocytes, B cells, T cells, and macrophages (14). Melanocytic carcinogenesis was also accompanied by immune cell infiltration. Researchers have further explored the low immune response in UM, revealing a reduction in the expression of the secreted phosphoprotein 1 signaling pathway gene in melanocytes, which leads to inadequate immune stimulation. In addition, the expression of the major histocompatibility complex class I pathway is increased in T cells, B cells, and macrophages, indicating immune dysfunction in the microenvironment.

Using SCS data from 17 patients with UM, researchers clustered 52,228 cells into 10 clusters (15). Among clusters 1, 3, 5, and 6, a high proportion of metastatic cells was observed. Cluster 5 revealed the most significant differences between primary and metastatic UM, with a high level of expression of GZMB, GPR183, and AREG, which are related to the immune response. In addition, cells in cluster 5 communicated frequently with other cells via the *IL10, SELPLG, EPHB,* and *ITGB2* signaling pathways, which may predict survival outcomes. A previous study analyzed the genomic profiles of two metastatic liver nodules, WL02 and WL03, in a patient with UM (16). Both samples were infiltrated by a high number of hepatic stellates and immune cells. Hepatic stellate and endothelial cells were abundant in WL02, whereas WL03 was enriched with T cells. Tumor cells in WL02 displayed a migratory tendency, with the enrichment of epithelial–mesenchymal transition, myogenesis, coagulation, and hypoxia genes, most of which were in an active proliferative state. These data demonstrate the complexity of UM liver metastases and highlight the importance of a precise assessment of heterogeneity when selecting the most suitable individual therapy for UM.

## 3 Tumor microenvironment

The TME consists of heterogeneous cells, including tumor cells and the surrounding non-neoplastic cells, such as immune, vascular, and fibroblast cell types. The interaction between tumor cells and their surrounding TME is related to the treatment response and prognosis in patients with malignancies (17). Previous studies have reported the existence of stromal cells in the TME of RB, specifically, retinal astrocytes that stimulate the proliferation of cone-like RB cells and macrophages that enhance RB progression (18–20). However, the molecular interactions between the TME and RB tumor cells are not yet fully understood. Wu et al. used SCS to study the effect of tumor and immune cells on the progression of RB (12). They found that the TME in RB consists of astrocyte-like tumor-associated macrophages (TAMs) and cancer-associated fibroblasts. During the tumor invasion process, the proportion of TAMs is reduced and M1-type macrophages are lost, which acts to suppress TAM-related immune functions and create an immunosuppressive microenvironment. TAMs self-regulate via the inhibition of the *CCL* and GALECTIN signaling pathways, while regulating tumor cells via the *GRN* and *MIF* signaling pathways. These findings provide new molecular insights into the TME of RB.

Accumulating evidence regarding the immune microenvironment has increased attention on pyroptosis, ferroptosis, and necroptosis as potential approaches for tumor management. Xie et al. integrated the SCS datasets of 11 UM samples and sorted the cells into low- and high-necroptosis groups according to the number of necroptosis genes in the individual cells (21). Using comprehensive bioinformatic technologies, they constructed the first necroptosis-associated prognostic model for UM. The high-necroptosis group exhibited stronger immune cell infiltration, including infiltration by M2 macrophages and T cells. Although a high necroptosis score is associated with poor prognosis, evidence to date regarding the value of certain immune checkpoint inhibitors for increasing therapeutic efficacy has been encouraging. Zhang et al. constructed a hypoxia-related prognostic model by integrating scRNA-seq and RNA-seq data (22). They depicted differences in the immune landscape of patients with UM between high- and low-risk groups. Their analyses revealed that follicular helper T cells, CD8+ T cells, activated natural killer (NK) cells, and gamma and delta T cells were significantly increased in high-risk groups, whereas naive B cells, memory resting CD4 T cells, resting mast cells, activated mast cells, resting NK cells, and monocytes were significantly increased in low-risk groups. Another study devised a prognostic model based on immune-related genes, including *S100A13*, *MMP9*, and *SEMA3B*, in which patients were divided into two groups (23). The TME landscape differed significantly between the two groups: antigen-presenting cells and dendritic cells were enriched in the high-risk group, whereas macrophage cells were more abundant in the low-risk group. Functional analysis revealed that knockdown of *S100A13* inhibited UM cell migration and proliferation, with an increase in reactive oxygen species-associated markers, whereas the reactive oxygen species pathway was most abundant in NK cells and platelet cells. These findings may help researchers identify new and efficient targets for immunotherapy.

BRCA1-associated protein 1 (BAP1) may be mutated in UM, leading to metastasis and a poor prognosis. Mutations in BAP1 have been identified in more than 80% of UM cases, and approximately 28% of patients with germline BAP1 alterations are diagnosed with UM, usually leading to metastasis within 5 years (24). Kaler et al. analyzed scRNA-seq data from 11 UM tumor samples and confirmed that BAP1 loss can lead to an increase in PROS1 expression in class 2 UM (characterized by poor prognosis and high metastatic risk) cells while increasing MERTK expression in CD163+ macrophages (25). This in turn stimulates macrophages into an anti-inflammatory M2-polarized state, following which M2-polarized macrophages secrete cytokines that suppress T cells and other types of immune cell. Ultimately, this generates an immunosuppressive microenvironment in UM tissue, which may be associated with therapeutic resistance to tebentafusp. Similarly, Figueiredo et al. found that the loss of BAP1 was related to suppressive immune responses, and that some genes containing *CD38, CD74,* and *HLA-DR* established an immunosuppressive axis following the loss of BAP1 (26). Single-cell analysis of five primary UM samples verified this hypothesis and revealed that infiltrating immune cells, including tumor-associated macrophages and regulatory CD8+ T lymphocytes, play a crucial role in generating the immunosuppressive TME in UM. Baqai et al. demonstrated that BAP1 loss in UM is associated with the upregulated expression of cell adhesion molecules (CADMs), such as E-cadherin, CADM1, and syndecan-2 (27). Comparing scRNA-seq data for BAP1 from wild and mutant type UM samples, researchers also reported that the upregulation of CADMS predominantly occurs in UM tumor cells. Interestingly, in the scRNA-seq data, two samples had cells that frequently expressed CADM1; however, not all BAP1 mutant cells exhibited increased levels of E-cadherin or CADM1. These findings provide new insights into the role of BAP1 in the development of UM.

## 4 Drug resistance

Chemotherapy is an important supplementary treatment for eye protection in patients with RB or UM, and slowing the emergence of chemical resistance remains the top treatment priority. Tumor heterogeneity is the main cause of tumor metastasis and drug resistance. Long-term and extensive use of chemotherapy drugs promotes tumor invasion and metastasis, which can occur easily in drug-resistant cells (28). Carboplatin is widely used to treat RB. A previous study explored the mechanisms of early resistance to carboplatin, which may derive from transcriptomic rearrangement via the PI3K/AKT signaling pathway, including metabolic adaption and the increase in transcription of the *ABCB1* transporter, instead of deriving from a minority of chemoresistant stem cells (29). Their results provide powerful evidence that can aid the development of pharmacological inhibitors, such as *ABCB1* transporter inhibitors, which can mitigate the emergence of drug resistance.

Using SCS technology, researchers have demonstrated that human melanoma cells are characterized by transcriptional variability. Such variability manifests as the infrequent and semi-coordinated transcription of drug resistance markers at a high level in some cells, which may aid in predicting which cells will develop drug resistance. These cells undergo epigenetic reprogramming after treatment with drugs and gradually develop a stable drug-resistant state (30). Rambow et al. demonstrated that the enrichment of neural crest stem cells, which are key drivers of drug resistance, in minimal residual disease in melanoma is mainly caused by *de novo* phenotypic transitions through transcriptional reprogramming, rather than by the enrichment of uncommon pre-existing cells (31).

Immune checkpoint inhibitor therapy has shown excellent efficacy for tumor treatment; nonetheless, recent studies have shown that programmed cell death protein 1 (PD1) and cytotoxic T lymphocyte-associated antigen-4 (CTLA4) blockades for the treatment of metastatic UM are ineffective (32). Using scRNA-seq V(D)J analysis, Durante et al. revealed clonally expanded T cells and/or plasma cells in tumor samples, indicating that tumor-infiltrating immune cells can mount a response and suggesting that poor tumor mutation may not be the only reason for the poor response of UM to checkpoint inhibitors (33). They also found that tumor-infiltrating immune cells in UM samples contained CD8+ T cells, which had not been previously recognized, and that they mainly expressed the checkpoint marker *LAG3* rather than *PD1* or CTLA4. LAG3, a soluble lymphocyte activation gene 3 protein, is an immune checkpoint inhibitor with a strong synergistic effect with PD-1 and may be a promising cancer treatment in the future (34). A previous study also noted that the combination of LAG-3 and PD-1 contributed to meaningful antidrug resistance and antitumor activity in metastatic UM; however, further clinical research is needed (35).

## 5 Conclusion and discussion

Owing to tremendous advancements in our understanding of RB and UM, strategies for local tumor control and the probability of salvaging the eyeball have vastly improved. However, metastasis remains common, representing a significant barrier to good prognosis (36). The TME affects genesis, progression, metastasis, and response to treatment and has been deemed a therapeutic target in various forms of malignancy. Cancer is a dynamically evolving disease, and tumor cells generally become spatially and temporally heterogeneous, which may be a major obstacle to treatment (37). SCS studies have provided new insights into the inherent complexity of RB and UM as well as their TMEs, which paves the way for early screening, personalized treatment strategies, and survival outcome prediction.

Tissue biopsy is the gold standard for establishing a diagnosis of cancer; however, tissue biopsy of intraocular malignancy is difficult and may increase the risk of extraocular dissemination. Liquid biopsy—a convenient, safe, and repeatable biopsy technology—is increasingly used to detect, analyze, and monitor various types of cancer, including RB and UM (38–40). Recent studies have demonstrated that the aqueous humor can be safely sampled during RB management, and analyses of tumor-derived cell-free DNA and DNA methylation in the aqueous humor can provide insights into the characteristics of RB (41–44). In previous studies, circulating tumor DNA in blood specimens and the aqueous humor as well as soluble human leukocyte antigen and angiopoietin 2 in the aqueous humor were identified as potential prognostic factors for UM (45–48). Researchers have also used SCS technology to study circulating cells in the cerebrospinal fluid, providing a new insight into the diagnosis and therapy of neurologic diseases (49). The combination of SCS and liquid biopsy may represent a new approach for the detection, monitoring, and evaluation of the risk of metastasis in RB and UM.

Despite the advantages of SCS, it has several limitations for the management of intraocular malignancies. First, a sufficient quantity of cells must be sampled to ensure that all cell types can be labeled. However, obtaining intraocular tumor tissue is more difficult than obtaining tissue from solid tumors at other anatomical sites, and there are often insufficient cells in aqueous humor samples. Second, the obtaining and storing of samples requires further refinement to reduce cellular injury. Third, although the cost of analysis per cell has been reduced to an acceptable level using the current system, the comprehensive sequencing price is extremely high for thousands of cell types in a sample, hindering its broad application in tumor research. Finally, SCS may provide a large amount of information, and to better understand the results and fully quantify biological variations, more advanced and convenient computational strategies are required.

In summary, SCS has provided a promising future for studying tumor heterogeneity and the TME, knowledge of which is crucial for improving our diagnostic methods, biomarkers, therapeutic strategies, and methods of predicting prognosis. The broad use of SCS may improve knowledge regarding intraocular tumors and promote the development of individual therapeutic regimens to prevent tumor metastasis and relapse while improving survival outcomes.

## Author contributions

R-lW, J-dZ, and YS designed and supervised the study. L-fH, PM, and C-hY wrote the manuscript. CH helped to conceptualize the manuscript. All authors contributed to the article and approved the submitted version.
